# Book Reviews

**Published:** 1876-10

**Authors:** 


### Book Reviews.

[NOTE.—All works reviewed in the pages of the CHICAGO MEDICAL JOURNAL AND EXAMINER may be found in the extensive stock of W. B. KEEN, COOKE & CO., whose catalogue of Medical Books will be sent to any address upon request.]

A TREATISE ON THE DISEASES OF INFANCY AND CHILDHOOD, by *J. Lewis Smith, M.D.*, Physician to the New York Infants' Hospital; Physician to the Catholic Foundling Asylum; Physician to the Protestant Infant Asylum; Clinical Lecturer Bellevue Hospital Medical College, etc., etc. Third edition—enlarged and thoroughly revised. Henry C. Lea. Philadelphia, 1876.

In 1869 the first edition of this book made its appearance. Although incomplete in many respects at the time, several of the more important diseases of children being unnoticed, it was well received by the profession. Prof. Smith's writings have been largely based "on clinical observations and the inspection of the cadaver." His connection with a number of childrens' hospitals and asylums have given him superior advantages in this respect, and some of the articles in his first edition were exhaustive; they have not been improved on by any author since.

Three years later a second edition was published, and the volume made more complete by the description of nearly twenty additional diseases, among which may be named diseases incidental to birth, rachitis, tuberculosis, scrofula, intermittent, remittent and typhoid fevers, chorea, paralysis, etc., etc. Many new formulæ were also introduced. And now, scarcely six years since the book first saw the light, a third edition, "enlarged and thoroughly revised," comes to us. Some of the old articles have been rewritten, and a few diseases, among which we notice rœtheln and cerebro-spinal fever, considered for the first time.

While it will he our purpose in this brief review to notice more particularly the newly described diseases, we can hardly refrain from speaking of the general plan of the work, a plan which has been adhered to throughout all the editions, and making an occasional comment on the more important subjects.

Part I. has chapters on the following subjects: 1, Infancy and Childhood; 2, Care of the Mother in Pregnancy; 3, Mortality of Early Life; 4, Lactation; 5, Selection of a Wet Nurse; 6, Course of Lactation — Weaning; 7, Artificial Feeding; 8, Baths — Clothing; 9, Accidents, etc., Incident to Birth and Detachment of the Cord; 10, Conjunctivitis Neonatorum; 11, Diseases of the Umbilicus; 12, Umbilical Hæmorrhage; 13, Diagnosis of Infantile Diseases.

It is not customary, we believe, for authors to include many of these subjects in works on diseases of children, but, put in Prof. Smith's style they are exceedingly valuable. His facts and rules in reference to lactation; his remarks in regard to modification of the milk in consequence of diet — from retention in the breast, by age, mental depression, catamenial function and pregnancy, are of great importance to every young practitioner.

Any brief consideration of so important a subject as artificial feeding must necessarily be unsatisfactory, but a very large amount of needful information has been crowded into the five pages devoted to this subject. The author agrees with Prof. Jacobi that those kinds of food which consist largely of starch are suitable for young babies, and the experiments of Dr. Korowin, of St. Petersburg, comparing the action of the pancreas and parotids whose secretion chiefly digests starchy food, have been repeated by Dr. Smith. It seems to be fairly established that but very small provision is made in the young infant for changing starch into sugar, and that the general belief of mothers, nurses and some physicians, where interference with the normal food of babies is demanded, that potatoe, bread crumbs, or any other substance containing starch, should be used either alone or with milk, is erroneous. Such food remains unchanged, it cannot be digested, it is good for nothing but to produce colic.

Section 1, Part II., is taken up with the consideration of diathetic diseases, four of which are mentioned, viz.: rachitis, scrofula, tuberculosis and syphilis. The second named disease is defined as characterized by increased vulnerability of the tissues, with a tendency to low grades of inflammation and hyperplasia of the lymphatics.

Eruptive fevers are considered under the next section.

The means by which scarlatina may be transmitted, and the extraordinary tenacity or permanence of the virus, are spoken of at considerable length. Neither of these subjects has received the attention, it appears to us, which its importance demands. Unless we doubt the truthfulness of such observers as Murchison, Louis, Thomas, Trousseau, and our author, it must be evident to all, that physicians, nurses and and attendants may, and frequently do, carry the contagion. Mason Good believes that a box of toys was, in one case, the means of transportation; and one of the authorities named above is confident that a letter and a lock of hair have been carriers of this dreadful poison. This contagion also, whatever it is, adheres so firmly to ordinary material, and resists with such absolute success the common exposure to air and the usual means of purification and cleanliness, that unusual care should be exercised if we expect to destroy its vitality. In the practice of Dr. Kearney Rogers, infection followed a visit to the apartments, three and a half months after the disappearance of of the disease in other patients. Richardson believes that the contagion became so fixed in the thatch of a house that infection followed more than five months after the occurrence of the first case, and Hildenbrand's coat is said to have retained the contagiousness one and a half years.

In regard to the treatment, especially of broncho pneumonia of measles, our author does not speak of what the Germans declare the most advantageous method, *i. e.*, hydrotherapeutics. In malignant forms of scarlet fever, however, with a temperature of 105° or higher, the antipyretic virtue of quinine is acknowledged and its employment advised. The cold affusions recommended by Trousseau, and whose testimony is that he never administered them without deriving some benefit, are regarded by Prof. Smith as liable to produce shock in young children, and perhaps other important and unfavorable complications.

The disease rœtheln is rare, perhaps new in this country. An epidemic of this peculiar malady has recently prevailed in New York city, and we are given the author's experience in it. A most interesting diagnostic syllabus of this disease, scarlet fever and measles, by one of the associate editors of this journal, having appeared in the April number, it seems superfluous to say more in regard to this affection. Among the authorities cited, the name of Prof. J. L. Smith appears frequently.

The most important disease considered in the next section, the *non-eruptive contagious* and embracing diphtheria, pertussis and parotiditis, is the first named. The author remarks in his preface that diphtheria has become a disease of great importance in this country, and * * * the most fatal malady of childhood in the localities where it prevails. Much study has evidently been given to this subject by Prof. Smith, and the article almost entirely rewritten. Great research has been made during the last few years concerning the nature of diphtheria, its cause, etc., etc. Not only have very careful clinical inquiries been made, but the microscope has been called to the aid of our investigators. The relation of *vegetable organisms to diphtheria* is now a very important question. Oertel claims that vegetable parasites throng all diphtheritic membranes, incisions, ulcers, etc.; fill the blood and lymph vessels; appear heaped up in the uriniferous tubules, inducing, in a way he explains, septicæmia and that long train of secondary affections so frightful in their near and remote results. The same author quotes Eberth — "without micrococci there can be no diphtheria." Oertel also claims that diphtheria is first always a *local* and then a *general* disease.

Prof. Smith, with his friend, Dr. Heitzmann, "a most excellent microscopist," have lately tried to verify the facts obtained by the German microscopists and pathologists. They find the bacteria, but in *non*-diphtheritic deposit as well as in true diphtheritic exudation. He says, page 221: "The micrococcus in the inflammatory product upon the fauces certainly does not indicate disease of a specific nature," and he believes that it is possible that Oertel and his advocates have mistaken a consequence for a cause. The treatment advised is not materially different from that which has given the profession in the West the best results, viz.: Tr. ferri chl., quinia, chlorate potass, internally; carbolic acid, liq. ferri subsulph., etc., as local applications.

In regard to the use of quinia in whooping cough, the author remarks that it has been prescribed for a considerable number of children in the Catholic Foundling Asylum, without diminishing materially the severity of the cough. It will be remembered that Dr. B. F. Dawson advocates very strongly the value of quinia in *curing* the *whooping* of this disease.

Of the general diseases mentioned in section four, and including malarial, typhoid and cerebro-spinal fevers, rheumatism and erysipelas, nothing special demands notice. The author believes, with other writers, that rheumatism is not as infrequent among children as commonly supposed. Many affections called by the people "growing pains," etc., have been rheumatic, and oftimes are followed with cardiac complications. "The disease at this age (in infants) may be so obscure, or latent, as to be overlooked even by good diagnosticians."—p. 307. If the article had been written in June of the current year, the author might have mentioned salicylic acid in the treatment. It appears to be of decided value in this disease.

Part III. occupies 147 pages of the book, and is devoted to diseases of the cerebro-spinal system. All the diseases usually included are here well described — the article on tetanus being particularly elaborate. Hydrate of chloral seems to be the remedy preferable, one physician stating that he has saved fifty per cent. since commencing its use. The tr. of calabar bean has also been praised by recent authors.

The next section, devoted to diseases of the respiratory organs, is exceedingly clear and very instructive. The article on pneumonia is especially good. The author prefers aconite to control the action of the heart, and believes it less depressing than veratrum viride. We have occasionally seen this effect from the veratrum, but when its action is closely watched by some one competent to count the pulse, it has given us the happiest results.

The pathological results of dentition are pointed out in the section given to diseases of the digestive apparatus, and the moderate use of the gum lancet suggested. Under the head of Indigestion, and when speaking of pepsin, he says: "Boudault's, from Paris, has been most used in this country, but the American preparations are probably equally good." When he prescribes it, he gives to an infant of three months, three grains combined with the same amount of subnitrate of bismuth. Query: Is pulv. *creb.* comp. c. opio a preparation peculiar to the East, or have we found an error?—p. 631, line 40.

The term cholera infantum is employed by our author "to designate only that form of infantile diarrhœa in which there are frequent watery stools, accompanied by vomiting, great elevation of temperature, and rapid and great emaciation." We earnestly commend this definition to a class of gentlemen in our midst professedly practicing a partial system of medicine, who always have an immense number of cases of cholera infantum, and none of simple diarrhœa and entero-colitis.

The consideration of cyanosis, and a few of the more important skin diseases, bring us to the end of the volume.

In conclusion, we have nothing but good words for Prof. J. Lewis Smith's book. Taking everything into consideration, we believe the present edition is the best work on children's diseases in the English language. C. W. E.

STATISTICS, MEDICAL AND ANTHROPOLOGICAL, OF THE PROVOST-MARSHAL-GENERAL'S BUREAU, DERIVED FROM RECORDS OF THE EXAMINATION for Military Service in the Armies of the United States, during the late War of the Rebellion, of one million Recruits, Drafted Men, Substitutes, and Enrolled Men. Compiled under the direction of the Secretary of War, by *I. H. Baxter, A.M., M.D.*, Colonel and Chief Medical Purveyor United States Army, late Chief Medical officer of the Provost-Marshal-General's Bureau. Government Printing Office.

These two large quarto volumes have been prepared under the supervision of Dr. I. H. Baxter, and contain so much valuable matter in so condensed a form that a very elaborate review would be necessary to express even generally the results of his analysis.

For the first two years of the war the enlistments were under the control of State authorities, but, this method proving inadequate, Congress, March 3d, 1863, created the Provost Marshal-General's Bureau, and ordered an enrollment of all persons liable to military duty. During the existence of this bureau four drafts were made, which furnished records of 605,045 examinations. The number excepted was 155,780, or a ratio of 257.39 per thousand. During the same period, 225,000 volunteers and 80,000 substitutes were examined; of the former a ratio of 221.63, and of the latter 264.17 per thousand were rejected. The plan of giving millesimal ratios is continued throughout the work, and makes the records easy of comprehension and comparison one with another.

The first volume opens with an introductory chapter, in which are detailed the recruiting regulations of the United States since the formation of the army, and those now in force in various foreign countries. The importance of comparing these regulations, and adopting the most stringent, is shown by the fact that in time of peace 7 per cent. of the fighting men of the English army are in the hospital, while in the peninsular war there were 21 per cent., and in the Crimean war 39 per cent. During our own war from 7 to 17 per cent., were incapacitated from service.

The last section of this chapter is devoted to anthropometry, and besides containing an excellent bibliography, has an extensive review of the whole subject. The total number of men credited on the several calls made by the President from the beginning to the end of the war, and put into actual service, was 2,762,401, and when the total rejected men is added, some idea may be formed of the opportunity afforded for the measurement and examination of the human body. The method of adopting some one portion of the human body as a modulus, and determining its remaining proportions by their supposed relation to this unit, is found to be almost invariably erroneous. But these variations arrange themselves in groups which follow numerically the law known as the law of the co-efficients of the binomial. The conclusions arrived at by the most eminent investigators in this branch of science are as follows:

1st. There is a perfect form or type of man, and the tendency of the race is to attain this type.

2d. The order of growth is regular towards this type.

3d. The variations follow a definite law, the law of accidental causes.

4th. The line formed by these variations is the curve known as the binomial.

A review of the tabular statements contained in the second volume follows, and the elementary conditions which enter into comparison with each other are: height, girth of chest, expansion of chest, age, weight, complexion, nativity, social condition and locality. The comparisons of each of these with the others, and a consideration of their relation to disease, may be stated as the scope of this report. The nomenclature of diseases adopted by the Royal College of Physicians and Surgeons in 1869, is employed with some modifications.

To determine the mean national stature, it is necessary first to decide as accurately as possible when man attains his full height; and the statistical results from over 190,000 examinations, showing the relation of height, girth and expansion of chest to age, indicate that the white natives of the Northern States do not attain full stature till between thirty and thirty-five years of age, while the Germans attain their full height at from twenty-five to thirty. The experience of other nations demonstrates the important bearing of this fact on the claims of the government for military service. In 1868, M. Champouillon, in the department of the Seine, had occasion to re-examine those who had been exempt in 1864, 1865 and 1866, and he found that of one hundred men who had been rejected in 1864 as below the standard, seventy-one had attained the requisite height in 1868; of the class of 1865, fifty-five men; and of the class of 1866, forty-five men.

A table showing the order of superiority in mean stature of twenty-four nativities, confirms the ethnological axiom that blond races are characterized by superior stature. The United

States Indian, with a mean average of 67.93 inches, stands at the head of the list; the United States whites are second; England, with an average of 66.57 inches, is eleventh; Germany twelfth, and France eighteenth, while Portugal is last of all, with an average of 65.43 inches.

An examination of the gradation in mean stature of the different States, places Kentucky, with an average of 68.67 inches, first, and Connecticut, with an average of 66.58 inches, last; the Western States generally having a higher mean average than the Eastern.

In measurements of the chest the man was naked, and the tape pressed evenly upon the nipples in front and the angles of the scapulæ behind, and the term "mean girth of chest," is to be understood as meaning at completed expiration. It was an interesting question to determine whether the development of the thorax increased in regular relation to increasing height, and such relation was not found to exist when comparing the mean height and mean girth of chest of all nations, or of the different States of the Union, but when each race is taken separately it is seen that the girth of chest increases as the height extends, with a regularity which would almost admit of a calculation by arithmetical progression in place of actual measurements. The records of the expansibility of the chest are extremely copious, and prove that the mobility of the chest also increases in arithmetical progression with increasing stature. Three inches was found to be a healthy mean expansion, but these records exhibit many instances of expansion of chest reaching seven inches. A remarkable instance of great expansive power was observed in a native of New Jersey, who was 28 years of age, 64 inches tall, 114 pounds in weight, and had an expansive power of seven inches. Among the cases of very limited mobility was that of a man who was 65½ inches tall, weight 125 pounds, and had an expansion of hardly half an inch. In 501,068 examinations of all nationalities, whose mean height was 67.30 inches, and mean circumference of chest 33.53 inches, there was a mean expansion of 2.78 inches.

We would naturally expect to find that the mean age of the volunteers would be lower than the foreign born, or men conscripted, and of 190,000 men accepted, one-fourth were under twenty years of age, while the mean age of the whole number was 26.24: Only a few returns of weight were made, but from 1,000 observations, the mean weight was 140.91 pounds, the maximum being 220 pounds, and the minimum 100 pounds.

The term military aptitude expresses the union of all the conditions of admissibility into military service, and has been much employed by European writers. It is a statement of the number in the thousand of young men of twenty who are found fit for the army. Our statistics do not admit of comparison with European nations, as men of all ages between eighteen and forty-five years were enrolled; but we find that of 5,020,942 men enrolled, 3,807,432 were found fit for service, which makes the military aptitude of the nation for all ages, 760 in the thousand.

The next part includes sixty charts and eleven tinted maps; the former picturing to the eye the most interesting results of the tables, by representing numbers by lines, which bear the same relation, in linear measurement, to each other as do the numbers for which they stand. Twenty-four charts show the relation of various diseases to social condition, complexion, age, height and nativity; ten, the relation of disease to occupation; twenty-four, the relation to locality (by States), and two charts exhibit the relation of both, height and girth of chest to age and nativity. The maps show, approximately, by gradation of color, the prevalence of certain diseases throughout that part of the United States wherein the drafts were enforced. The charts show that married men were, as a rule, much more affected than the single; that men of light complexion were found more affected than the dark; that, as a general rule, the older men furnished a larger ratio of rejected, and lastly, that the ratio of rejections increases with the increase of height.

Of all nativities the Indians of the United States were found the most healthy, while Mexico suffered more from disqualifying diseases than any other country. Something remarkable is the close gouping of the English speaking countries, as we find in the list of twenty-four nationalities, arranged in the order of healthfulness, that England stands No. 12, and the United States (white) No. 13, while Ireland, Wales and Scotland are Nos. 10, 11 and 14, respectively.

In studying the charts in relation to disease and occupation, we observe a steady and regular increase of disease as we ascend the social scale from the unskilled laborer to the professional man. Certain occupations which have generally been considered very unhealthy are shown not to be so on examination; for instance, the tobacconist and liquor dealer are found to be more healthy, as regards their digestive and nervous systems, than the dealers in other merchandise, and in fact than the average for all occupations. Lawyers are more subject to disease than men of any other profession, while editors suffer most from phthisis pulmonalis and more than the average with diseases of the digestive system. The musicians are shown to be the healthiest as a class.

The third and last part consists of reports of surgeons of the board of enrollment, and gives their judgment upon the fitness and sufficiency of the enrollment laws, together with a graphic account of the physical characteristics and the social and hygenic condition of the inhabitants of their respective districts. The portions of the report most interesting to the medical profession, are the accounts of the prevailing diseases of each district, with the accompanying explanation of the etiology; but the special value of these reports lies in the fact that they furnish a comprehensive view of the nosography of the Northern States, taken simultaneously by competent observers.

The first volume closes with an exhaustive and well arranged analytical index. An edition of five thousand copies of this work has been published, and its distribution is reserved to members of the present congress. H. H.

The Chicago Medical Register. For 1876–7. Published under the supervision of the Chicago Medico-Historical Society. D. W. Graham, A.M., M.D., Editor. Chicago: W. T. Keener, Publisher. 1876.

This most commendable publication appears this year in a more tasteful style than it did a year ago — with cloth covers and fine heavy paper — and it appears earlier. It is a little book of twenty-eight pages, and in the present edition all matter has been excluded except the list of regular physicians and of the officers of the society. The make-up of "the list" is sufficiently indicated by the title, which reads as follows: "Physicians in Chicago who have been accredited to the Chicago Medico-Historical Society as in good and regular standing according to the Code of Ethics of the American Medical Association, and whose names are liable to excision from the list for violation of the Code." The list contains three hundred and fifty-three (353) names, and it probably more nearly approaches the ideal of what the society would make it than has any previous edition.

The Medico-Historical Society is to be congratulated on the persistent and very sensible way in which it has prosecuted its work, and — in this age of fallible men and women — on results with so few imperfections as it this year presents. The editor is to be especially congratulated.

The list of officers is as follows: President, Thos. Bevan; Vice-President, E. L. Holmes; Treasurer, R. G. Bogue; Secretary, P. S. Hayes; Diarist, S. Wickersham. Publication Committee: E. Ingals, John Bartlett, F. C. Hotz.

